# Concurrent Recording of Co-localized Electroencephalography and Local Field Potential in Rodent

**DOI:** 10.3791/56447

**Published:** 2017-11-30

**Authors:** Sungmin Kang, Michael Bruyns-Haylett, Yurie Hayashi, Ying Zheng

**Affiliations:** ^1^School of Biological Sciences, Whiteknights, University of Reading; ^2^Department of Bioengineering, Imperial College

**Keywords:** Neuroscience, Issue 129, Local field potential, electroencephalography, event related potential, concurrent recording, burr hole, co-localize, barrel cortex, whisker stimulation, rodent

## Abstract

Although electroencephalography (EEG) is widely used as a non-invasive technique for recording neural activities of the brain, our understanding of the neurogenesis of EEG is still very limited. Local field potentials (LFPs) recorded via a multi-laminar microelectrode can provide a more detailed account of simultaneous neural activity across different cortical layers in the neocortex, but the technique is invasive. Combining EEG and LFP measurements in a pre-clinical model can greatly enhance understanding of the neural mechanisms involved in the generation of EEG signals, and facilitate the derivation of a more realistic and biologically accurate mathematical model of EEG. A simple procedure for acquiring concurrent and co-localized EEG and multi-laminar LFP signals in the anesthetized rodent is presented here. We also investigated whether EEG signals were significantly affected by a burr hole drilled in the skull for the insertion of a microelectrode. Our results suggest that the burr hole has a negligible impact on EEG recordings.

**Figure:**
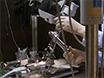


## Introduction

It is generally accepted that LFPs recorded via microelectrodes primarily reflect the weighted sum of synchronized excitatory and inhibitory synaptic activities of local pyramidal neural populations^1^,^2^,^3^,^4^. Our recent research demonstrated that the profile of the LFP signal could be separated into components of excitation and inhibition^5^,^6^. However, as LFP is normally measured via an invasive procedure, it is not suited for most studies of the human brain.

On the other hand, EEG is a non-invasive technique for measuring the electrical activity of the brain. It is widely used as a diagnostic tool for certain types of neurological diseases such as epilepsy, and as a research tool in human cognitive studies. Despite its popularity, a major limitation of EEG is the inability to interpret its temporal profiles precisely in terms of the underlying neural signals^7^,^8^,^9^.

Increasingly, mathematical models of EEG are developed to enhance understanding of brain function^10^,^11^,^12^,^13^,^14^,^15^. Most of the existing EEG models are developed based on fitting frequency domain characteristics of the model predicted output to the EEG data spectrum during spontaneous activity, and very few EEG models can generate realistic sensory evoked potentials. In this context, concurrent recordings of EEG and LFP will provide important insight and constraints for developing more accurate mathematical models of EEG.

To address this need for concurrent recordings to further explore the neural origin of EEG, we developed a methodology to simultaneously record EEG and multi-laminar LFP signals in the neocortex of the anesthetized rat. The setup is similar to previous concurrent EEG/LFP studies conducted in primates^16^,^17^. We further investigated the effect of a burr hole drilled into the skull on EEG recordings surrounding the hole, by comparing bilateral EEG recordings (*i.e.*, one hemisphere with a burr hole, the other hemisphere intact) in the absence of sensory stimulation. Our results demonstrate that concurrent EEG/LFP recordings can be conducted simply and effectively, with little EEG signal distortion from the burr hole in the skull.

## Protocol

All experiments were carried out in accordance with the British Home Office regulations (Animals (Scientific Procedures) Act, 1986) and approved by the Research Ethics Committee at the University of Reading, UK.

### 1. Animal Preparation

NOTE: Female Lister Hooded rats were used for all experiments. This is a non-survival procedure. 

1. Record the rat's weight on a laboratory scale.
2. Anesthetize the rat in a chamber with 5% isoflurane and an oxygen flow rate of 1 L/min.
3. Place the rat onto a stereotaxic holder with a paper towel underneath its body and with its teeth resting via the bite bar.. The paper towel will make the insertion of a heat pad easier (see step 2.3) and catch any excrement from the rat during the experiment.
4. Administer isoflurane continuously via a hard-plastic nose cone mounted onto the nose clamp for rat adaptor at a concentration of 3% with an oxygen flow rate of 0.5 L/min. Connect the cone to a small animal isoflurane anesthetic system.

### 2. Surgical Procedure

1. Insert a thermostatic heating pad underneath the paper towel upon which the rat is resting, secure the rat’s head with two ear bars, and monitor the body temperature using a rectal thermometer.
2. Shave the top of the rat's head.
3. Apply ophthalmic ointment to the eyes to prevent corneal drying.
4. Before exposing the cranium, apply lidocaine drops to the scalp and massage it gently into the skin.
5. Make a midline incision of approximately 2-3 cm on the scalp using a scalpel to expose the surface of the skull.
6. Carefully separate the temporalis muscle contra-lateral to the whisker pad to be stimulated from the skull using a Jacquette Scaler and a pair of serrated and curved dissecting forceps. Clean the skull with cotton swabs whenever necessary.
7. Using a braided silk, non-absorbable suture, tie the separated muscle to the scalp with a tight knot and then tie the suture securely to the stereotaxic frame^18^.
8. Use stereotaxic coordinates to locate the barrel cortex, 2.5 mm caudal to bregma and 6 mm lateral to midline^19^. Draw a dot at the location of the somatosensory cortex using a pencil or a marker.
9. **Drill a burr hole at the marked location using a dental drill. To prevent the skull from overheating during drilling, apply sterile saline (sodium chloride 0.9%) to the work area every 10-15 s. The drilling process involves the following 3 steps:**
  1. Drill a hole of diameter < 2 mm into the skull using a drill bit #4 (0.055 in diameter). Take care not to drill into the dura.
  2. Thin the bottom of the hole to translucency using a drill bit #1/4 (0.019 in diameter).
  3. Use a 27 G needle to pierce the dura to allow the insertion of a microelectrode.
10. Transfer the rat, secured on a stereotaxic frame, to a Faraday cage mounted on top of a vibration isolation workstation.
11. Attach an oximeter sensor clamp connected to an oximeter control unit to the rat's hind paw to monitor continuously the following physiological parameters: heart rate, breath rate, arterial oxygen saturation, pulse distention, and breath distention. These parameters were displayed continuously on a PC monitor, reflecting the physiological condition and anaesthetic depth of the rat.
12. Replace the hard-plastic nose cone for isoflurane administration and the nose clamp for rat adaptor with a microflex breather fitted with a transparent soft nose cone which is modified (Figure 1A) to allow easy whisker stimulation to one side of the whisker pad without compromising the isoflurane administration.
13. Insert two stainless steel stimulating electrodes to the whisker pad exposed by the cut-out on the nose cone.
14. Connect the stimulating electrodes to an isolated current stimulator.
15. Lift the skin of the midline of the neck with forceps and make a 1~2 cm incision with scissors ready for the placement of reference electrodes. Take care not to cut the muscle tissue.

### 3. Co-localized EEG/LFP Setup

1. Clean and dry the skull surrounding the burr hole using a cotton swab.
2. Carefully place the conductive EEG paste on one flat side of an EEG spider electrode. Leave a small hole clear of the EEG paste on the spider electrode to allow a multi-laminar microelectrode to pass through the hole without contacting the paste and the spider electrode. This prevents electrical contact between the EEG electrode and the microelectrode.
3. Align the spider electrode to the burr hole in the skull, with the EEG paste facing the skull.
4. Carefully press the spider electrode onto the skull, making firm contact with the skull via the EEG paste. Remove any paste obscuring the burr hole using a needle on a syringe.
5. Remove excessive EEG paste beyond the periphery of the spider electrode so that the contact between the spider electrode and the skull is spatially constrained to the size of the electrode (Figure 1B).

**Figure:**
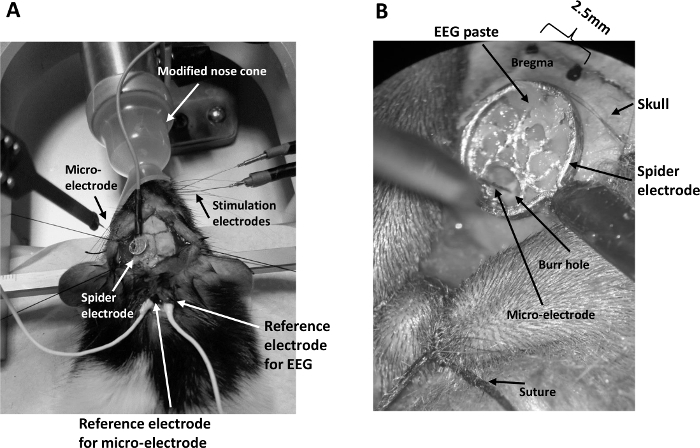

**Figure 1: General setup for concurrent EEG/LFP recording.** (**A**) The setup consists of a modified nose cone for ease of whisker pad stimulation under isoflurane anesthesia, two stimulating electrodes inserted into the whisker pad, a spider electrode positioned on the skull above the barrel cortex contra-lateral to the stimulating electrodes, a multi-channel microelectrode inserted into the barrel cortex through the spider electrode, and reference electrodes placed inside an incision at the back of the rat's neck. (**B**) A view through the microscope of the spider electrode securely positioned onto the skull by EEG paste. The microelectrode is inserted into a burr hole drilled into the skull under the spider electrode. The scalp is held back by surgical thread (suture) tied to the stereotaxic frame. Please click here to view a larger version of this figure.

1. Smear EEG paste onto the reference electrode for the EEG and place it securely inside the incision at the back of the rat’s neck.
2. Connect the EEG electrodes to the preamplifier via a passive signal splitter for low impedance signals (Figure 2). Make sure the impedance of the spider electrode is below 5 kΩ. If it is not, check that the EEG paste is in good contact with the skull and the electrode is firmly pressed to the EEG paste. Add more EEG paste if necessary.
3. Mount a micromanipulator arm on the stereotaxic frame. Connect a linear 16-channel microelectrode (100 µm spacing, area of each site 177 µm^2^) to a 16-channel acute headstage clipped securely onto the micromanipulator arm.
4. Smear EEG paste onto the reference electrodes for the EEG and microelectrode, then securely place them inside the incision (Figure 1A).
5. Adjust the angle of the micromanipulator arm so that the microelectrode is perpendicular to the cortical surface. This angle is normally between 25-35 °.
6. Lower the microelectrode under a microscope by turning the micromanipulator knobs so that the tip of the microelectrode is aimed at the tiny opening at the bottom of the burr hole until the uppermost electrode just penetrates the cortical surface. Care must be taken to avoid forcing the microelectrode onto the surface of the dura as this would break the electrode.
7. Couple the 16-channel microelectrode to a preamplifier connected to a data acquisition unit via a fiber optic cable (Figure 2).
8. Turn on the preamplifier, the data acquisition unit, and the computer connected to the unit. Turn on the stimulator box.
9. Insert the microelectrode normally to the cortical surface by slowly turning the z-axis knob of the micromanipulator to a depth of 1,500 µm^20^.
10. Micro-adjust the depth by applying a train of stimulus to the whisker pad and observing the 16-channel evoked LFP on a PC monitor using the software of the data acquisition unit installed on the PC. Carefully turn the z-axis knob on the micromanipulator until the highest amplitude of the evoked LFP occurs around channel 7 (as this coincides with layer IV in the cortex). NOTE: Ipsi-lateral EEG electrode setup: For some experiments, a second spider electrode was placed on the ipsi-lateral side of the intact skull above the barrel cortex. This setup allowed bilateral EEG recording during the resting state to investigate the effect of the burr hole on the EEG signal. NOTE: The surgical procedure to set up the EEG electrode is identical to that described above, except that during step 2.6, the temporalis muscle on each side of the head was carefully separated from the skull, sutured back and tied securely to the corresponding side of the stereotaxic frame. NOTE: The concurrent EEG/LFP setup is also identical to that described above, with an additional step that a second spider electrode is loaded with the EEG paste, then pressed firmly to the skull above the ipsi-lateral barrel cortex.

**Figure:**
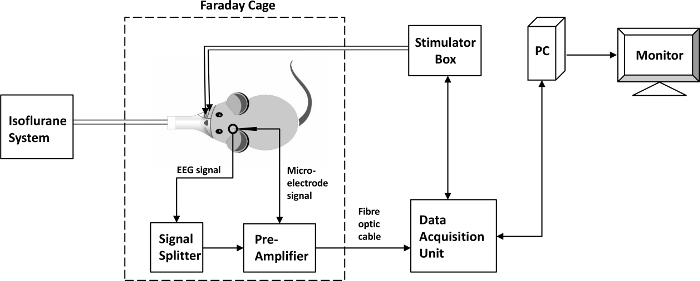

**Figure 2. A signal flow diagram.** The rat is placed inside a Faraday cage. The stimulating electrodes receive commands from the stimulator box controlled by the Data Acquisition Unit through its software installed on a PC. The neural signal recorded by the microelectrode is transmitted to a pre-amplifier inside the Faraday cage. The neural signal recorded by the EEG probe is transmitted to the pre-amplifier through a signal splitter. The pre-amplifier is connected to the Data Acquisition Unit outside the Faraday cage via a fiber optic cable. The neural data are then stored on a local drive on the PC, while they can also be displayed on a PC monitor. A mobile small animal isoflurane system administers isoflurane from outside the Faraday cage. Please click here to view a larger version of this figure.

### 4. Electrical Stimulation and Neural Recordings

NOTE: The sampling frequency for all neural data is 24.41 kHz with 16-bit resolution. A trial consists of a single electrical stimulation at the start of the trial. Each trial lasts 10 s, which is also the inter-stimulus interval (ISI). Each stimulus is a square current pulse of 1.2 mA lasting 0.3 ms. For bilateral experiments to study the effect of the burr hole, continuous resting state of 250 s is also recorded.

1. Open the recording software on the computer in use.
2. Load the correct circuit for the experiment by selecting 'Load Project…' from the dropdown menu of 'OpenProject'. A new window ('WorkBench') will appear (Figure 3).

**Figure:**
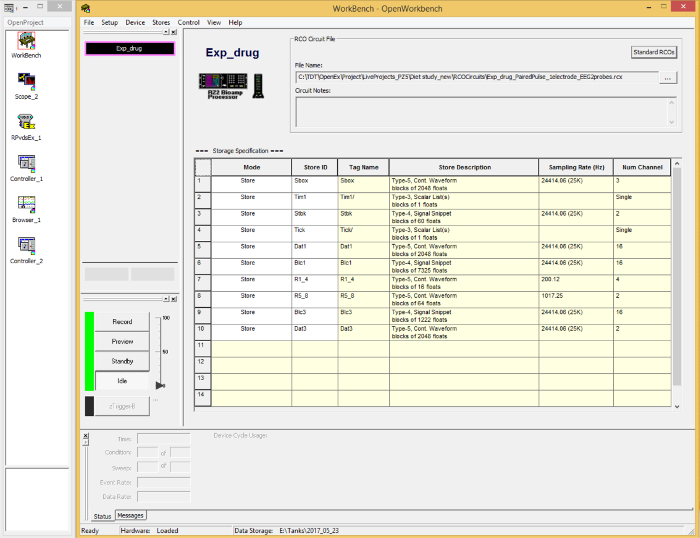

**Figure 3. A display of the software GUI for the Data Acquisition Unit.** It allows the appropriate circuit to be uploaded, stimulation parameters to be set, and data to be recorded and visualized. Please click here to view a larger version of this figure.

1. **Create a new directory (called a 'Tank' by the software) to store neural recordings.**
  1. Click on 'File' from the top of the window, and select 'Data Task Management'. A new window ('Tank Management') will appear.
  2. In the 'Tank Management' window, press the right button of the mouse to display a menu. Select 'Create New Tank'. Another new window ('Create Data Tank') will appear.
  3. In the 'Create Data Tank' window, select the path where you plan to create a new data directory, and enter the name of the new directory. Then press 'OK'. This window will disappear.
  4. The new directory will appear in the 'Tank Management' window but in grey. Register this directory by right-clicking on it, and select 'Register Tank' from the dropdown menu. A red star and a green arrow will appear to the left of the new directory's name which is now in black (Figure 4).
  5. Unregister any previous directories not in use by right-clicking in the 'Tank Management' window and selecting 'Refresh Tank List' in the dropdown menu.
  6. Click on 'OK' to exit the 'Tank Management' window.

**Figure:**
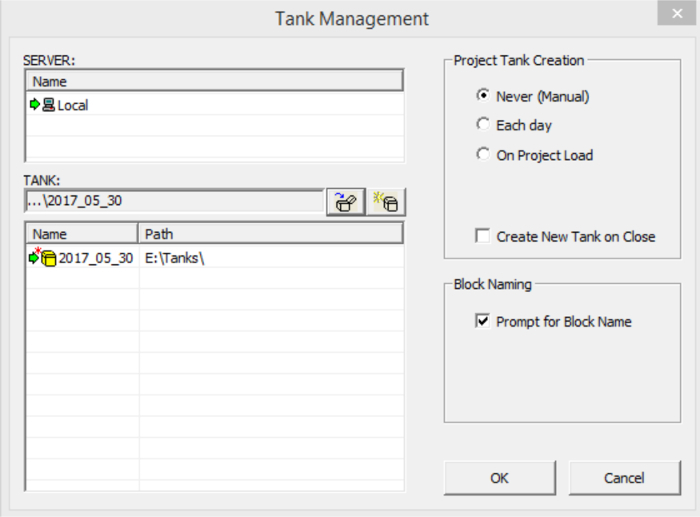

**Figure 4: A display of the software GUI showing a registered data directory.** Please click here to view a larger version of this figure.

1. **Register the new directory in 'Scope' to display neural signals during experiment.**
  1. Click on the 'Scope' icon in the 'OpenProject' window. A new window ('Scope') will appear.
  2. Right click the mouse in the 'Scope' window and select 'Refresh Tank List' in the dropdown menu. The new directory's name will appear in grey.
  3. Click on the new directory. A red star and a green arrow will appear to the left of the new directory's name which is now in black.
2. Set up the experimental parameters for data acquisition in the 'WorkBench' window by clicking on 'Setup' from the top of the window. A new window will appear. Select 'Sweep loop', set the length of the trial and the number of trials to be recorded.
3. Check that the Stimulator Box is turned on.
4. Press the 'Record' button in the 'WorkBench' window. A new window will appear. Enter the name of the data file you want to save for the experimental run but do not hit the return button at this stage, as the EEG recording parameters need to be set up.
5. **Set up the EEG recording parameters using the Graphic User Interface (GUI) on the pre-amplifier. Touch the screen (anywhere) of the preamplifier to wake up the screen. Select 'Unlock' to unlock the display (Figure 5).**
  1. Press the left icon in the '2: EEG' panel. A new display will appear.
  2. Press 'Coupling' and select 'AC'.
  3. Press 'Ref Mode' and select 'Local'.
  4. Press 'Samp Rate' and select '25KHz'.
  5. Press 'OK' to return to the original display.

**Figure:**
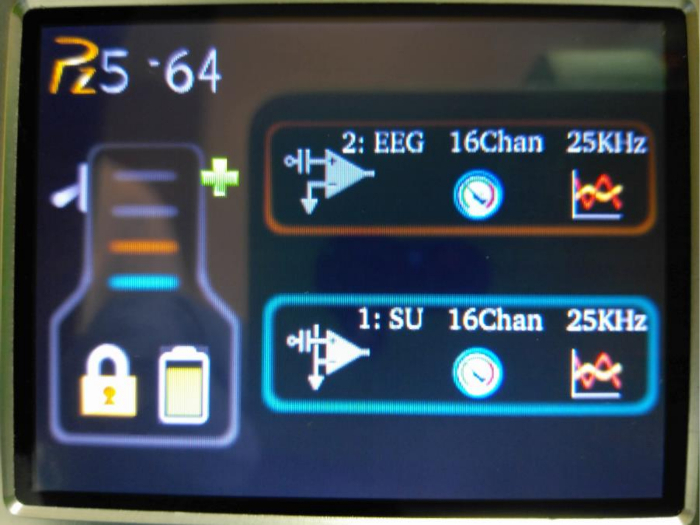

**Figure 5: The GUI on the pre-amplifier.** It allows EEG recording parameters (*e.g.*, sampling frequency and referencing preference) to be set. Please click here to view a larger version of this figure.

1. Check the impedance of the EEG probe(s) by pressing the middle icon in the '2: EEG' panel. If too high, add more EEG paste to the probe. Press 'OK' to return to the original display.
2. Wait 20 s to avoid recording the initial fluctuation of the EEG recordings.
3. Go back to the PC monitor (after the 20 s wait) and press the 'Return' key on the keyboard. The EEG and the LFP signals will be recorded.

### 5. Data Analysis

1. **Pre-process the evoked LFP and EEG signals on a trial-by-trial basis using the following steps.**
  1. Shift back the neural data in time by 20 samples (equivalent to 0.82 ms). This is the delay produced by the circuit used to collect neural data in TDT itself. By shifting the data, the time zero point is aligned to the onset of the stimulus.
  2. Remove the stimulus artifact by replacing the neural data from 0 to 1 ms with a straight line connecting the data point at 0 ms with the data point at 1 ms.
  3. Zero-mean each trial by subtracting the mean value of the neural signal 200 ms prior to stimulus onset.
  4. Low-pass filter the data below 800 Hz using a 4^th^ order Butterworth IIR type filter in both directions to avoid introducing any temporal shift in the data.
  5. Align the multi-laminar data across animals. For each animal's LFP data, apply the inverse Current Source Density (spline iCSD, source radius R = 0.5 mm) analysis^21^ with a Gaussian filter (λ = 50 µm) to locate the layer IV sink^1^, which is given by the largest negative peak occurring at a cortical depth below the pial surface within the first 15 ms of stimulus onset. The CSD, and the corresponding LFP, data are then aligned according to their sink locations across animals. The common sink is located in layer IV, ~ 600 µm below the pial surface.
  6. After alignment, use channels 2, 7, and 12 of the realigned LFP as representatives of neural responses of the supragranular, granular, and infragranular layers, respectively in the barrel cortex.
2. Calculate the mean evoked LFP and EEG by averaging the pre-processed data over 100 trials.
3. To investigate the effect of the burr hole on the EEG, down-sample the EEG signals to 1,000 Hz, and compute the power spectral density (PSD) for the contra-lateral (with a hole in the skull) and ipsi-lateral (intact skull) spider electrode recordings over a 250 s period of resting state. PSD is computed from 0.1-100 Hz in Matlab using the function 'pmtm' which is based on the multitaper method^22^.
4. Divide the frequency range into the following well-known frequency bands: Delta (δ): 0.1-4 Hz, Theta (): 4-8 Hz, Alpha (α): 8-13 Hz, Beta (β): 13-31 Hz, Gamma (γ): 31-100 Hz. Calculate the average PSD within each band.
5. Within each frequency band, calculate the normalized difference in PSD, *P_err_*, between the contra- and the ipsi-lateral EEG using the equation: 
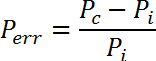
 where *P_c_* and *P_i_* are the average PSD of the contra- and ipsi-lateral EEG, respectively in the frequency band of interest.
6. Within each frequency band, perform a one-sample t-test to test the hypothesis that there is no significant difference (at 0.05 significance level) between the PSD of the EEG signal recorded from the two hemispheres.

## Representative Results

Data from 4 rats were averaged to obtain mean time series where applicable. The amplitude of the evoked EEG response, also known as the event related potential (ERP), is typically much smaller than that of the LFP. Figure 6 shows the average ERP and LFP in the supragranular, granular, and infragranular layers of the barrel cortex, respectively. The error band in each plot is the corresponding standard error. It can be seen that ERP is approximately 10 times smaller than the evoked LFP.

**Figure:**
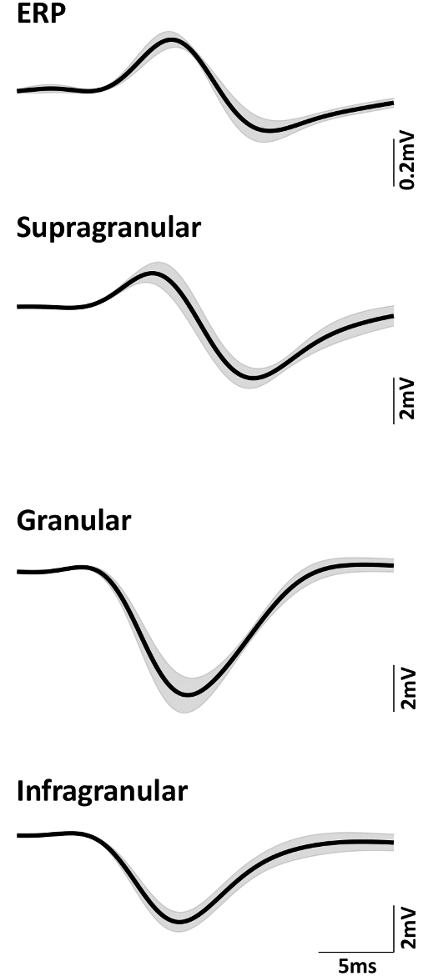

**Figure 6: Mean (n = 4) neural signals of ERP, supragranular, granular, and infragranular LFP.** Shadow indicates standard error. Please click here to view a larger version of this figure.

Comparisons of the temporal dynamics of ERP and LFP are shown in Figure 7. Direct superposition of ERP and the supragranular LFP in Figure 7A illustrates the order of amplitude differences between these two types of neural signals. To compare the temporal dynamics, both ERP and LFP are normalized with respect to their negative peak amplitude. Figure 7B and **7C** show the normalized ERP superimposed with the normalized supragranular LFP and normalized granular LFP, respectively.

It can be seen from Figure 7B that the peaks of P1 and N1 for ERP are more delayed than the corresponding peaks of LFP in the supragranular layer. However, the temporal profiles of these two neural signals are similar, with P1 preceding N1. On the other hand, the temporal profile of ERP is markedly different from that of the granular (layer IV of the barrel cortex) LFP (Figure 7C). Importantly they are not mirror images of each other, with granular LFP dominated by a single negative peak (reflecting a major sink in cortical layer IV), whereas ERP consisted primarily of two peaks with opposite polarity.

**Figure:**
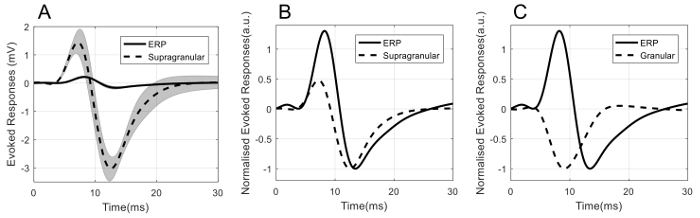

**Figure 7: Comparison of the temporal dynamics of ERP and LFP.** (**A**) ERP (solid line) superimposed with supragranular LFP (dashed line). Shadow indicates standard error. (**B**) Normalized ERP (solid line) superimposed with normalized supragranular LFP (dashed line). (**C**) Normalized ERP (solid line) superimposed with normalized granular LFP (dashed line). Please click here to view a larger version of this figure.

The ERP signal was measured via a spider electrode placed on the skull with a burr hole drilled into it. To investigate the effect of the hole on EEG recordings, another spider electrode was placed on the intact skull above the ipsi-lateral barrel cortex. Care was taken to ensure that the impedances of the two spider electrodes were comparable in magnitude by adjusting the amount of EEG paste used. Data from four rats (which were not the same rats used above) are presented here.

Figure 8 shows the concurrent resting state EEG recordings from both electrodes of one rat, with 100 s data displayed in Figure 8A, and the data in the rectangular frame (20 s) are expanded in Figure 8B. The two EEG signals largely co-vary, within similar range of amplitude. Figure 9 shows the PSD of the four rats, with the top row using a linear scale on the frequency axis, and the bottom row using a logarithmic scale on the frequency axis to provide an expanded view in the lower frequency range. From Figure 9, there does not appear to be consistent bias in the PSD across subjects. This was confirmed by performing one-sample t-tests on the normalized differences in the averaged PSD in the five frequency bands, shown in Figure 10. None of the normalized PSD differences in these frequency bands were significantly different from zero (p = 0.32, 0.46, 0.85, 0.69, and 0.97, respectively).

**Figure:**
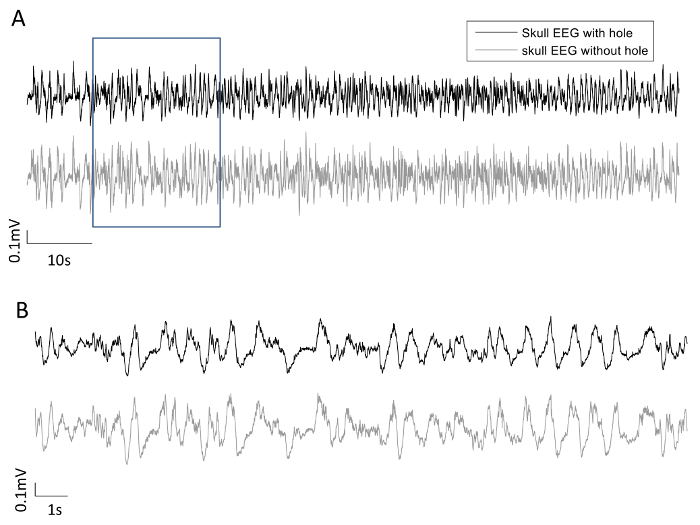

**Figure 8: Bilateral EEG recordings.** (**A**) Skull EEG recording during resting state with a burr hole in the skull (black) and a simultaneous EEG recording on the opposite hemisphere with the skull intact (grey). (**B**) Expanded view of the waveforms within the rectangular frame in (**A**). Please click here to view a larger version of this figure.

**Figure:**
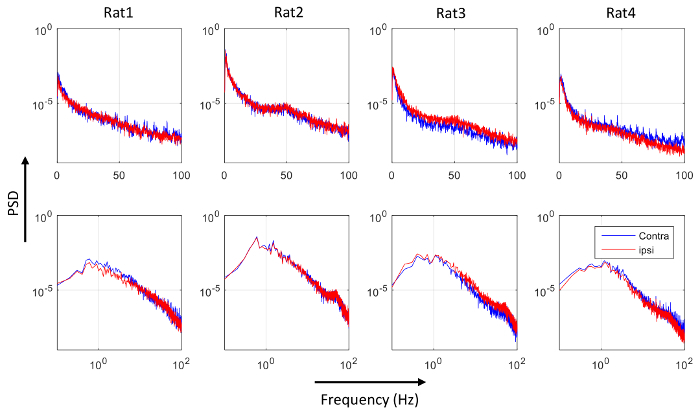

**Figure 9: Power spectral density (PSD) of the contra- (blue) and the ipsi-lateral (red) EEG.** Each column shows the PSD for one rat. The top panels use linear frequency scale, while the bottom panels use logarithmic frequency scale to allow the PSD in the lower frequency range to be visualized. Please click here to view a larger version of this figure.

**Figure:**
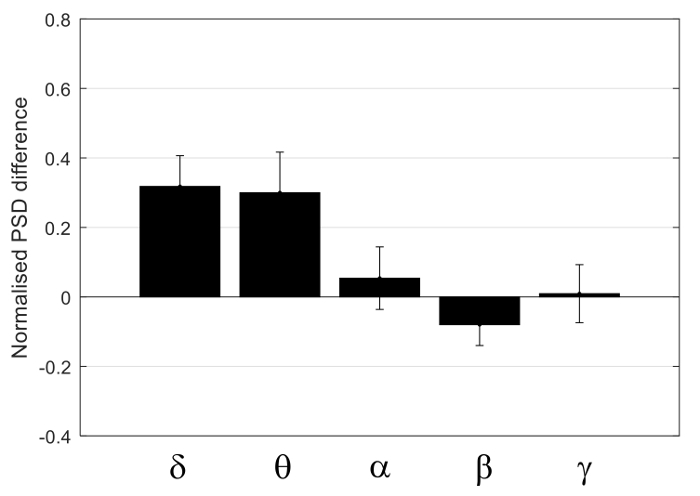

**Figure 10: Group analysis.** Normalized difference between the contra- and the ipsi-lateral PSD with the five frequency bands: Delta, Theta, Alpha, Beta, and Gamma. Each bar shows the mean normalized differences within the frequency band, with the standard error shown as the error bar. Please click here to view a larger version of this figure.

## Discussion

We have described an experimental procedure for concurrent recording of co-localized EEG and LFP signals of an isoflurane anesthetized rat in response to whisker pad stimulation. A microelectrode was inserted into the neocortex through an opening in the EEG spider electrode which was aligned with a burr hole drilled into the skull. The electrode was secured to the skull by a conductive and adhesive EEG paste^23^. The nose cone used for the administration of isoflurane was modified so that stimulating electrodes could be inserted into the whisker pad with ease.

The EEG paste was effective at mounting the spider electrode securely to the skull, while providing excellent electrical conductivity throughout the experimental day without the need for additional application of paste. It replaced the undesirable use of glue to fix the periphery of the spider electrode to the skull, as glue is non-conductive and can increase the impedance of the electrode if it runs between the skull and the electrode. EEG paste has a number of advantages over EEG gel, which is difficult to shape around the burr hole and can dry out during experiment, resulting in poor EEG signals.

As the rat was placed inside a Faraday cage, electrical noise due to the environment was greatly attenuated. However, sometimes the neural signal was still quite noisy. In most cases, this was caused by the reference electrode not securely positioned and therefore needed to be re-adjusted or more EEG paste used. Another common problem was that the evoked LFP was small in amplitude. This could be due to the microelectrode not positioned at the center of the cortical region activated by the stimulating electrodes. Instead of re-inserting the microelectrode, which could cause more damage to the local neurons, we usually adjusted the position of the stimulating electrodes in the whisker pad until a reasonable amplitude of the LFP (>3 mV) could be observed.

One of the limitations of the technique is the poor spatial resolution of the spider electrode, which has a diameter of 6 mm. This is large compared with the size of the rat's skull. Unfortunately, the spider electrode used here is the smallest available to purchase. It will be desirable to reduce the diameter of the spider electrode to 2-4 mm, thus increasing the spatial specificity of EEG recordings, making the comparison between the EEG signal and the supragranular LFP signal less ambiguous.

Several critical steps in the protocol need special attention. The first is the insertion of the microelectrode through the burr hole. As the dura is otherwise intact, the precision of the insertion is crucial. A slight resistance at the tip of the electrode usually means the electrode is not positioned correctly. It must be raised, position adjusted, and re-inserted. The second is the position of the nose cone on the rat. It must not be too loose, as isoflurane will escape from the cone. It also must not be too tight, as this can obstruct the nostrils of the rat and cause difficulty breathing. Special attention is also required to ensure that the amplitude of the EEG recording is much smaller (usually 5 to 10 times smaller) than the LFP top channel recording. If they are similar, it is an indication that the EEG probe has come into direct or indirect contact with the microelectrode. An indirect contact is usually through the cerebral spinal fluid (CSF) that sometimes fills the hole drilled in the skull. The conductivity of CSF is typically 100 times that of the skull^24^,^25^. Thus, if the level of CSF inside the burr hole is sufficiently high, it can make contact with the spider electrode. To avoid this, the hole should be frequently cleaned with super absorbent cotton sponges such as the absorption spears.

The effect of a burr hole (diameter <2 mm) in the skull on the EEG recording surrounding the hole was studied by placing another spider electrode on the intact skull atop the ipsi-lateral barrel cortex so that bilateral EEG recordings could be compared. The results shown in Figure 9** and **Figure 10, suggest the effect to be insignificant at the 0.05 level of significance. Other factors affecting the amplitude of the EEG include how well the EEG paste was in contact with the skull, how firm the electrode was pressed to the paste, and the spatial extent of the EEG paste on the skull.

It is also worthwhile to note that the protocol described here recorded skull EEG, which is different from scalp EEG used in human EEG studies. The scalp acts like a resistor or a low-pass filter, which will reduce the signal-to-noise ratio of the EEG recording further.

Finally, comparison of the temporal dynamics of the ERP and that of the evoked LFP across cortical layers suggest that somatosensory evoked potential reflects better the LFP in the supragranular layer of the cortex than that in the granular and infragranular layers. This is in agreement with our earlier work^6^, demonstrating that the initial segment (P1) of the ERP is related to the return current arising from the inflow of the excitatory synaptic current occurring in the granular layer, while the subsequent decrease (N1) in ERP may be related to the delayed arrival of thalamic afferent to cortical layers II/III and/or feedforward signals from deeper cortical layers. In conclusion, concurrent recordings of EEG/LFP can enhance understanding of the neural genesis of EEG, and facilitate the mathematical modeling of EEG in terms of neural signals across cortical layers.

## Disclosures

The authors have nothing to disclose.
